# Cardiac surgery in patients with Hemophilia:is it safe?

**DOI:** 10.1186/s13019-020-01123-0

**Published:** 2020-05-08

**Authors:** Amjad Shalabi, Erez Kachel, Alexander Kogan, Leonid Sternik, Liza Grosman-Rimon, Ronny Ben-Avi, Diab Ghanem, Eyalon Ram, Ehud Raanani, Mudi Misgav

**Affiliations:** 1 Chaim Sheba Medical Center, Tel-Hashomer, 52621 Ramat-Gan, Israel; 2 Affiliated to the Sackler School of Medicine, Tel Aviv University, Tel Aviv-Yafo, Israel; 3 Cardiovascular Department and Research Center, Poriya Medical Center, 15208 Tiberias, Israel; 4 affiliated to the Azrieli Faculty of Medicine, Bar-Ilan University, Safed, Israel; 5 The National Hemophilia Center, Chaim Sheba Medical Center, Tel-Hashomer, 52621 Ramat-Gan, Israel

**Keywords:** Cardiac surgery, Hemophilia a, Hemophilia B, Factor XI deficiency, Cardiovascular disease

## Abstract

**Background:**

The life expectancy of hemophiliacs is similar to that of the general population. As a result, the prevalence of age-related cardiovascular diseases has increased. We present our experience with hemophilia patients who underwent cardiac surgery in our Medical Center between 2004 and 2019.

**Methods:**

All hemophilia patients who underwent cardiac surgery were identified, and their peri-operative data evaluated retrospectively.

**Results:**

Ten patients were identified: six with hemophilia-A, one with hemophilia-B, and three with hemophilia-C (factor XI deficiency). Cardiac procedures included ten coronary artery bypass grafts and one aortic valve replacement. Hemophilia-A and B patients were treated with factor substitution, whereas patients with factor XI deficiency were treated with fresh frozen plasma. One patient died, and one patient suffered from non-active gastrointestinal bleeding.

**Conclusions:**

While major cardiac surgery can be performed safely on patients with hemophilia, a multidisciplinary team approach and strict postoperative monitoring are essential in order to achieve optimal results.

## Background

Hemophilia comprises a group of inherited bleeding disorders caused by low concentrations of specific coagulation factors. Hemophilia-A and B are hereditary X-linked bleeding disorders caused by a deficiency or absence of coagulation factors: VIII or IX, respectively. Factor-XI deficiency, originally called hemophilia-C, is less common, and occurs primarily among Ashkenazi Jews. In contrast to hemophilia-A and B, there is no relationship between the risk of bleeding and the extent to which there is a factor-XI deficiency.

Recently, there have been remarkable strides in the diagnosis and treatment of hemophilia patients, especially regarding the development of safer and newer generations of factor concentrates. As a result, the life expectancy of these patients has increased, bringing it in line with that of the general population [1]. However, with this increase of life expectancy, more hemophilia patients are confronted with age-related co-morbidities including: ischemic heart disease and degenerative valves which necessitate surgical intervention [2, 3]. The deficiency of coagulation factors in hemophilia patients may provide a protective effect from thrombus formation but does not influence the mechanism of atherosclerotic development [4]. This explains why hemophilia patients are not protected against arterial cardiovascular disease. In a recent study, Miesbach, et al. found that approximately 30% of patients over the age of sixty with moderate or severe hemophilia were diagnosed with cardiovascular diseases [5].

Cardiac surgery is associated with coagulation abnormalities leading to an increased intra- and post-operative bleeding risk secondary to heparinization, surgical trauma, extracorporeal circulation, hypothermia, and increased fibrinolysis [4, 5]. Therefore, cardiac surgery in patients with hemophilia is extremely challenging since hemostasis has to be balanced with anticoagulation. The literature provides only sparse information regarding the results of cardiac surgery among patients with hemophilia. The results of our study, which present the outcome of hemophilia patients who underwent cardiac surgery in our Department, could shed further light on this patient cohort.

## Methods

### Study cohort

A total of ten patients with hemophilia underwent cardiac surgery at the Sheba Medical Center between 2004 and 2019. Six patients had hemophilia-A, one patient had hemophilia B and three patients had hemophilia C (factor XI deficiency). The diagnosis of hemophilia was carried out by clinical hematologists. All cardiac procedures were performed with cardiopulmonary bypass. **The Indications for surgery was according to the accepted guidelines and are similar to those without hemophilia- typically, three vessel coronary arterial disease, stenosis of the left main and symptomatic valve disorders** [6]. All patients were well known to the National Hemophilia Center in our Medical Center.

### Surgical procedures

In total, we performed eleven cardio-surgical procedures: ten coronary artery bypass grafts (CABG), and one concomitant case with an aortic valve replacement.

### Surgical technique

Surgery was performed through a full sternotomy. Standard cardiopulmonary bypass (CPB) was established by cannulation of the ascending aorta and the right atrium. Myocardial protection was achieved by using cold blood cardioplegia. Heparin was given before and during the CPB in order to achieve an activated clotting time (ACT) greater than 450 s. Protamine sulfate was administered at the end of the operation to achieve a normal value of ACT. After completing anastomosis, de-airing was performed carefully, the cross clamp was removed, and the patient was weaned from CPB. A cell saver (C.A.T.S; Fresenius AG, Bad Homburg, Germany) was used in all patients for autologous blood transfusion at the end of the operation.

### Classification of hemophilia severity

The severity of hemophilia is based on factor levels. In hemophilia A/B, the factor level of < 1% is classified as severe; 1–5% as moderate, and > 5% as mild. For factor XI deficiency any level below 20% is considered severe.

### Hemostasis protocol

#### Hemophilia -a and -B patients

The appropriate factor concentrate was given 1 h before induction. Patients with a deficiency in factor VIII received 45 IU/kg; patients with factor IX deficiency received 100 IU/kg. After weaning from the machine, IV boluses of the appropriate factor concentrate were administered (~ 2000 IU). Management of the factor-infusions was overseen by an experienced hematologist in the operating room. We checked the level factor at two and 4 h after the operation and then twice daily, in the morning and evening. Recently, we have started to use thromboelastography (TEG) to assess hemostasis peri-operatively [7].

#### Hemophilia-C (factor-XI deficiency)

Infusion of fresh frozen plasma (FFP) was used in order to achieve factor levels of 30–40 Udl^− 1^. After weaning from the machine, patients received FFP according to the activated partial thromboplastin time (APTT). The decision to treat those patients with FFP was left to the discretion of the hematologist. We checked APTT twice daily in the morning and evening.

## Results

### Clinical data

Clinical data are listed in Table 1. Six patients had hemophilia-A (3 with mild, 2 with moderate and one patient with severe hemophilia-A). One patient had hemophilia-B, and three patients had severe factor-XI deficiency, with a level below 20%. Risk factors for ischemic heart disease were found in 80% of the patients. All patients underwent CABG: one patient underwent concomitant aortic valve replacement. Two patients (20%) had hepatitis-C and three patients (30%) had a positive family history of hemophilia. The mean left ventricular ejection fraction was 54.4 ± 5%.

**Table 1:** Clinical data

	Hemophilia A and B							Hemophilia C (Factor XI deficiency)		
ID	A	B	C	D	E	F	G	H	I	J
Gender	M	M	M	M	M	M	M	F	M	M
Age (years)	65	38	70	57	74	71	61	58	62	67
Hemophilia	A	A	A	A	B	A	A	C	C	C
Type	Severe^●^ > 1%	Mod^●^ 5%	Mod^●^ 3%	Mild^●^ 8%	Mild^●^ 9%	Mild^●^ 7%	Mild^●^ 8%	Severe > 20%	Severe > 20%	Severe > 20%
Risk factors	Yes	No	Yes	Yes	Yes	Yes	Yes	Yes	Yes	No
HCV	No	No	No	Yes	Yes	No	No	No	No	No
HIV	No	No	No	No	No	No	No	No	No	No
FH of hemophilia	Yes	Yes	No	Yes	No	No	No	No	No	No
Surgery	CABG	CABG	CABG	CABG	CABG+AVR	CABG	CABG	CABG	CABG	CABG
EF%	55	56	50	65	55	48	52	62	58	50
EuroScore%	1	0.5	1.1	0.7	1.1	0.9	1	0.6	0.7	0.7

*M* Male; *F* Female; *HCV* Hepatitis C; *HIV* Humans immune deficiency virus; *FH* Family history; *EF* Ejection fraction; *Mod* Moderate; *CABG* Coronary artery bypass grafting; *AVR* Aortic valve replacement. Mild*= > 5% of procoagulant level; Moderate^●^ = 1–5% of procoagulant factor; Severe^●^ = < 1% of procoagulant level.

### Transfusion and factor replacement

Patient D, who died 4 days after discharge, received twelve units of packed red blood cells (PRBCs), eight units of FFP, and six units of platelets (PLTs) on the day he died. Three patients required post-operative transfusions. In addition, two patients (A and C) each received only one unit of PRBCs intra-operatively. Three patients (A, C and E) each required one unit of PRBC post-operatively. All patients with hemophilia-C received FFP before the operation. Patient G received FFP and PLTs post-operatively. None of the hemophilia C patients received PRPCs or PLT transfusions.

We do not intend to report the amount of factor consumption, since we believe that hemophilia patient management is highly individualized. Such details do not provide any instructive or effective information.

### Clinical outcomes

Clinical outcomes are listed in Table 2. One patient died on the 12th post-operative day due to antibody development against factor 8, resulting in bleeding and tamponade. One patient was readmitted due to upper gastrointestinal bleeding. Gastroscopy did not show any active bleeding. The patient was treated with proton pump inhibitors. There was no need for a PRBC transfusion. Wound infection was not observed in any of the patients. None of the patients developed factor antibodies during the 6 months following surgery.

**Table 2:** Clinical outcomes

	Hemophilia A and B							Hemophilia C (Factor XI deficiency)		
ID	A	B	C	D	E	F	G	H	I	J
Mortality	No	No	No	Yes	No	No	No	No	No	No
Renal failure	No	No	No	Yes	No	No	No	No	No	No
Wound infections	No	No	No	No	No	No	No	No	No	No
Re-exploration	No	No	No	Yes	No	No	No	No	No	No
Antiplatelet on discharge	Yes	Yes	Yes	Yes	Yes	Yes	Yes	Yes	Yes	Yes
Antibodies	No	No	No	Yes	No	No	No	No	No	No
GI Bleeding	No	No	No	Yes	Yes	No	No	No	No	No
FFP	No	No	No	Yes	No	No	Yes	Yes	Yes	Yes
PRBC	Yes	No	Yes	Yes	Yes	No	No	No	No	No
Platelet	No	No	No	Yes	No	No	Yes	No	No	No
**Amount of chest drains (cc)**	**380**	**290**	**300**	**270**	**420**	**320**	**400**	**300**	**280**	**230**
**Chest drain removal (day)**	**3**	**2**	**3**	**2**	**4**	**3**	**4**	**3**	**2**	**2**

*GI* Gastrointestinal bleeding; *FFP* Fresh frozen plasma; *PRBC* Packed red blood cells;

### Comparison with non-hemophilic patients who underwent CABG

As seen in Table 3, extracorporeal circulation and aortic cross clamp time of the hemophilia patients were similar to those of the non-hemophilia patients who underwent CABG in our Medical Center during the past 5 years. The length of hospital stay between hemophilia and non-hemophilia patients was also comparable.

**Table 3:** Comparison between hemophilia and non-hemophilia patients who underwent isolated CABG during the past 5 years

	Hemophilia (***n*** = 8)	Non-hemophilia (***n*** = 1716)	***P*** value
ECC duration (mins)	89 ± 15	92 ± 36	0.7
ACC duration (mins)	56 ± 12	62 ± 41	0.4
LOS in ICU (days)	2 ± 4	3 ± 24	0.9
LOV (hours)	8 ± 3	14 ± 28	0.5
Hospital duration (days)	14 ± 4	8 ± 19	0.3

*ECC* Extracorporeal circulation; *ACC* Aortic cross clamp; *LOS* Length of stay; *ICU* Intensive care unit; *LOV* Length of ventilation.

### Our management consensus

Table 4 summarizes our protocol treatment for hemophilia patients requiring cardiac surgery.

**Table 4:** Management consensus for individuals with hemophilia

Preoperative assessments	Classification of the disease Multidisciplinary team: surgery should be performed in hospitals that have a hemophilia treatment center Inhibitor measurements (Bethesda assay)
Intra-operativetreatment	The factor level should be maintained above 80% at the end of surgery Factor transfusion should be managed by an experienced hematologist in the operating room Routine use of cell saver Routine use of TEG to monitor coagulation, especially during the process of heparin titration Antifibrinolytics during surgery Tissue valve should be preferred Maintain the factor level above 80% in the early 48 h post-operatively.
Post-operativetreatment	Continues is better than bolus factor replacement Factor measurements twice daily in the morning and evening Factor level replacement above 50% after 48 h Inhibitor screening if clinically indicated Intensive physiotherapy treatment and early mobility to prevent thrombosis Low-dose aspirin prophylaxis for life

## Discussion

The revolution in the treatment of hemophilia has resulted in a near normal life expectancy, and as a result hemophilia patients are now more likely to be confronted with age-related cardiovascular conditions such as ischemic heart disease and degenerative heart valves. Hemophilia patients are even more susceptible to certain traditional cardiovascular risk factors such as hypertension and obesity, due to limited mobility, secondary to arthropathy [8–11]. Furthermore, reduced factor levels do not seem to provide protection against the development of atherosclerosis [12].

Cardiac surgery on hemophilia patients is considered to be an extremely hemostatic challenge. Coagulopathy, particularly caused as a result of total heparinization and extracorporeal circulation, raises the bleeding risk. Our study showed that major cardiac surgery can be accomplished safely in patients with congenital factor deficiency. Unfortunately, there is a lack of evidence-based guidelines regarding the treatment of hemophilia patients who are eligible for cardiac surgery. Most current recommendations for hemostatic management in hemophilia patients are based on case reports and the opinion of experts. A standardized hemostatic treatment protocol seems to be an unlikely solution, based on the small number of hemophilia patients who undergo cardiac surgery, and the span of hemophilia severity. Currently, the best approach is to individualize the treatment protocol and introduce a policy of multidisciplinary teamwork.

The main objective of our treatment protocol was to reach almost normal factor levels before surgery and during the peri-operative period. On the morning of surgery, a bolus of factor concentrate was given to enable safe and bloodless anesthesia procedures as well as venous harvesting. During the immediate post-operative period we preferred to use continuous infusion of factor VIII, as originally described by our National Hemophilia Center [13]. Continuous infusion of factor VIII reduces total factor use, improves safety with stable activities of factor VIII, and guards against a lack of deep troughs below the hemostatic range [12]. **All of our hemophilia patients received tranexamic acid intraoperatively. TXA is an antifibrinolytic agent, acts as adjuncts in hemophilia patients due to reduction of the fibrinolytic activity** [14]**. We usually don’t give antifibrinolytics agents postoperatively, except in cases of bleeding despite optimal factor replacement therapy.**

Inhibitor development is one of the most significant complications in patients with hemophilia. Inhibitors appear less frequently, in approximately 3–13% of patients with mild or moderate hemophilia [15]. Inhibitors are classified into low- or high-responding based on the titer. A titer of 5BU differentiates between low and high responding inhibitors [16]. Despite the improvement in hemostatic agents, such as activated prothrombin complex concentrate (aPCC) or recombinant factor VIIa (rFVIIa), patients with inhibitors are still at increased risk of bleeding [17]. In 2009, patient D underwent an appendectomy. A few days later he developed an intra-abdominal hemorrhage despite appropriate factor VIII treatment because of inhibitor development. He was treated with activated prothrombin complex concentrate. Prior to CABG surgery, the level factor 8 and antibodies were 8 and 0%, respectively. He was readmitted on post-operative day twelve due to deterioration in his hemodynamic state. He was diagnosed with bleeding which resulted in cardiac tamponade and underwent urgent intervention. No clear source of bleeding was seen, but it was more a diffuse bleeding. We started treatment with rFVIIa (NovoSeven, Novo Nordisk, Bagsvared, Denmark), but without clinical response and the patient died. Of note, the patient developed inhibitors against factor 8 on the 10th post-operative day (7BU). For hemophilia patients with inhibitors, rFVIIa should be used. However, rFVIIa does not appear to be ideal treatment for patients without inhibitors, when FVIII and FIX concentrates are available, since they have a longer half-life and better clinical response [18].

One patient with factor XI deficiency, who underwent CABG, was diagnosed with aortic dissection type A 3 years after the procedure. In the initial operation the patient was treated with FFP, and in the second operation, was also treated with FFP which produced satisfactory hemostasis.

The current literature is sparse regarding the need for long-term anti-thrombotic treatment in hemophilia patients after cardiac surgery. Adopting the guidelines from the general population to hemophilia patients could be associated with increased bleeding risk. However, several case delineations reported safe anti-thrombotic treatment in hemophilia patients [19, 20]. According to the Dutch Group [21], anti-thrombotic recommendations should be the same as those adopted in the general population, on the condition that there is adequate replacement therapy. All of our study patients received antiplatelet therapy post-operatively. There is still a lack of data regarding the appropriate dose and duration of antiplatelet and anticoagulation therapy. Therefore, close cooperation between all the specialists is essential in order to optimize treatment and minimize the risk of bleeding.

This study has some limitations. It included a small number of heterogenic patients, and was also a retrospective, single-center study.

## Conclusions

Major cardiac surgery can be performed safely in patients with hemophilia. A multidisciplinary team approach and strict post-operative monitoring are essential in order to achieve optimal results.

## Data Availability

Not applicable.

## Abbreviations

ACT: Activated clotting time
aPCC: Activated prothrombin complex concentrate
APTT: Activated partial thromboplastic time
CABG: Coronary artery bypass graft
CPB: Cardiopulmonary bypass
FFP: Fresh frozen plasma
PLT: Platelets
PRBC: Packed red blood cells
rFVIIa: Recombinant factor VIIa
TEG: Thromboelastography
