# Correction to: Cardiac surgery in patients with Hemophilia:is it safe?

**DOI:** 10.1186/s13019-020-01186-z

**Published:** 2020-06-29

**Authors:** Amjad Shalabi, Erez Kachel, Alexander Kogan, Leonid Sternik, Liza Grosman-Rimon, Ronny Ben-Avi, Diab Ghanem, Eilon Ram, Ehud Raanani, Mudi Misgav

**Affiliations:** 1 Chaim Sheba Medical Center, Tel-Hashomer, 52621 Ramat-Gan, Israel; 2 Affiliated to the Sackler School of Medicine, Tel Aviv University, Tel Aviv-Yafo, Israel; 3 Cardiovascular Department and Research Center, Poriya Medical Center, 15208 Tiberias, Israel; 4 affiliated to the Azrieli Faculty of Medicine, Bar-Ilan University, Safed, Israel; 5 The National Hemophilia Center, Chaim Sheba Medical Center, Tel-Hashomer, 52621 Ramat-Gan, Israel

**Correction to: J Cardiothorac Surg 15, 76 (2020)**

**https://doi.org/10.1186/s13019-020-01123-0**

The original article [1] presents co-author, Eilon Ram’s name incorrectly as ‘Eyalon Ram’. The correct presentation is noted in this Correction article.
